# Raman-Based Diagnostics of Biotic and Abiotic Stresses in Plants. A Review

**DOI:** 10.3389/fpls.2020.616672

**Published:** 2021-01-20

**Authors:** William Z. Payne, Dmitry Kurouski

**Affiliations:** Department of Biochemistry and Biophysics, Texas A&M University, College Station, TX, United States

**Keywords:** digital farming, non-invasive phenotyping, nutrient content assessment, plant disease diagnostics, Raman spectroscopy, optical sensing

## Abstract

Digital farming is a novel agricultural philosophy that aims to maximize a crop yield with the minimal environmental impact. Digital farming requires the development of technologies that can work directly in the field providing information about a plant health. Raman spectroscopy (RS) is an emerging analytical technique that can be used for non-invasive, non-destructive, and confirmatory diagnostics of diseases, as well as the nutrient deficiencies in plants. RS is also capable of probing nutritional content of grains, as well as highly accurate identification plant species and their varieties. This allows for Raman-based phenotyping and digital selection of plants. These pieces of evidence suggest that RS can be used for chemical-free surveillance of plant health directly in the field. High selectivity and specificity of this technique show that RS may transform the agriculture in the US. This review critically discusses the most recent research articles that demonstrate the use of RS in diagnostics of abiotic and abiotic stresses in plants, as well as the identification of plant species and their nutritional analysis.

## Introduction

As the global population grows exponentially, the expansion of agricultural territories is restricted by a scarcity of rich land, an increase in cost, and operational time consumption of conventional farming. This problem can be solved by an expansion of agricultural territories or by the development of digital farming. While the first approach is destructive and inefficient, the second strategy is focused on an enhancement of the farming efficiency. By other means, digital farming, or precision agriculture, aims to maximize the crop yield with minimal environmental impact. This can be achieved by timely detection and identification of biotic (plant diseases) and abiotic [drought and nutrient deficiency (ND)] stresses.

Plant diseases caused by fungi and viruses can reduce the crop yield on average by 40%, depending on a host, the pathogen and environmental conditions (Mantri et al., 2012; Waqas et al., 2019). Confirmatory diagnosis of such diseases can be used for the precise application of fungicides and pesticides, allowing for highly efficient pathogen treatment, maximization of the crop yield and minimization of the environmental impact of farming (Farber et al., 2019a). There are several molecular and imaging techniques that can be used to detect biotic stresses (Raza et al., 2015). For instance, polymerase chain reaction (PCR) and enzyme-linked immunosorbent assay (ELISA) are commonly used for confirmatory diagnostics of plant diseases (Alvarez and Lou, 1985; Li et al., 2006; Lievens et al., 2006). Rapid development of these technologies enabled on-site, rather than laboratory-based, use of these methods (Ahrberg et al., 2016, 2020; Thomas et al., 2019). However, relatively high cost of PCR analysis (~$15 per sample) limits broad use of this technique in farming. Confirmatory diagnostic of abiotic stresses, such as nutrient deficiencies and drought, is far more challenging than the detection and identification of plant diseases. These conditions are also far more detrimental than a pathogen-induced stress: lack of nutrients, water or hyper salinity can cause up to a 70% reduction in the crop yield (Mantri et al., 2012; Waqas et al., 2019). There are several imaging techniques, such as hyperspectral imaging and thermography, that potentially can be used for an indirect detection of abiotic stresses in plants (Bauer et al., 2019; Caballero et al., 2020). These techniques allow for fast imaging of broad field areas and identification of “problematic areas” (Baena et al., 2017; Lu et al., 2020). However, these methods do not always possess required specificity. Diagnostics of nutrient deficiencies can also be achieved by the use of sophisticated chromatographic and colorimetric procedures (Zhu et al., 2008; Mihaljev et al., 2015), which are time and labor consuming. This catalyzes a search for alternative methods of diagnostics of plant stresses that can be inexpensive, fast, portable, and confirmatory.

Digital farming also requires advanced methodologies in plant breeding and selection (He et al., 2020; Wang et al., 2020). This is necessary to develop the germplasm of crops to have higher drought or soil salinity tolerance, as well as enhance the resistance to pathogens. One of the major drawbacks of conventional plant selection and breeding techniques is the long period of time that takes to measure the effect of a specific stress on plants (He et al., 2020; Wang et al., 2020). For example, the current *in vivo* techniques are focused on determination of physiological changes or plant chlorophyll contents, which are not directly related to the stress response and therefore require many experiments to draw meaningful conclusions (He et al., 2020). Biochemical *in vitro* techniques are more relevant but are destructive and labor-intensive. Because of unpredictable weather patterns, drought or fungal tolerance screening of breeding populations during the entire growing session over many months are difficult to perform as drought stress is difficult to control (Gao et al., 2008). To speed up this research, there is an urgent need to develop more robust phenotyping techniques for non-destructive, accurate and rapid assessment of breeding populations for drought-related responses, especially at the early seedling stages and with short periods of withholding water.

Raman spectroscopy allows for non-invasive and non-destructive detection and identification of biotic (Egging et al., 2018; Farber and Kurouski, 2018; Sanchez et al., 2019a, 2019c) and abiotic (Altangerel et al., 2017; Sanchez et al., 2020b) stresses. RS can be used for accurate and rapid plant phenotyping and the assessment of the nutritional content of grains (Krimmer et al., 2019; Farber et al., 2020c). RS is based on a phenomenon of inelastic light scattering by molecules that are being excited to higher vibrational or rotational states. After the first experimental demonstration of this phenomenon in 1928 by C. V. Raman, the spectroscopy of inelastic light scattering or RS continuously gain popularity in a large variety of research fields that range from food chemistry (Almeida et al., 2010) and electrochemistry (Zeng et al., 2016) to forensics (Kelly Virkler and Lednev, 2009; López-López et al., 2013) and materials science (Cantarero, 2015). Agriculture and farming, together with a basic plant biology, plant breeding, and pathology are relatively new unchartered territories for RS. One can expect that RS had far-reaching implications in agriculture broadly defined due to its non-invasiveness, non-destructiveness, high sensitivity, and a label-free nature. Raman had desired portability, low labor, and cost requirements (Yeturu et al., 2016; Farber and Kurouski, 2018; Farber et al., 2019a). Raman had no difficulty in scanning an entire orchid due to its quick analysis time (typically 1 s per reading) and high specificity for both biotic and abiotic stresses. The fast results of RS allow farmers to take advantage of the information and make quick adjustments to cease the development of a certain biotic or abiotic stress. The non-labor-intensive and non-destructive nature of Raman also allows for rapid assessment of the plant phenotype directly in the field, eliminating the need of a wet-laboratory analysis of plants (Krimmer et al., 2019; Farber et al., 2020c).

## Instrumentation and Imaging Approaches

**Figure 1:**
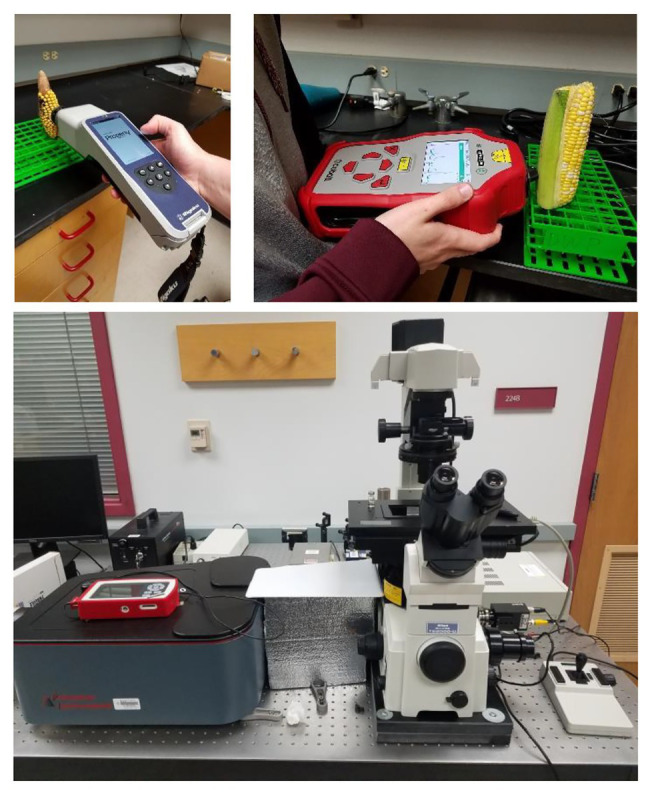
Two commercially available hand-held Raman spectrometers with 1,064 nm (left) and 830 nm (right) excitations (top) and a bench-top home-built confocal Raman microscope (bottom).

Although the instrumental concept of RS was known since 1928, rapid growth of this technique took place after the invention of lasers in 1960s and CCDs in 1980s (Cardona, 1975). Massive lasers used in first Raman spectrometers not only needed a large footprint of a laboratory space for such instruments but also required highly efficient water chillers. Appearance of solid-state continuous wavelength (CW) lasers and highly stable CCDs allowed for substantial militarization of Raman spectrometers. Currently, several companies offer excellent hand-held devices that can be used directly in the field or a crime scene (Figure 1). Although portable spectrometers continuously gain popularity, confocal Raman microscopes remain the instrument of choice if low amount of material is available or spatial resolution of the Raman measurements is required.

**Figure 2:**
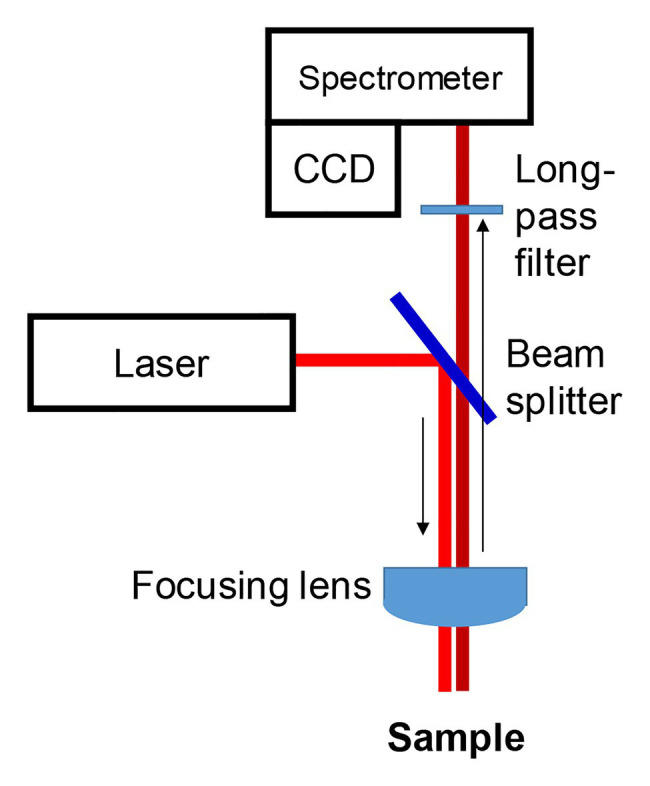
Schematic representation of a Raman spectrometer.

From a hardware perspective, confocal Raman microscopes and hand-held instruments share similar engineering concepts. Electromagnetic radiation generated by a laser source is directed by a beam splitter toward the sample. Achromatic lens or a microscope objective is then used to focus a light on the sample (Figure 2). Scattered light is collected typically using the same optical setup. Next, with a use of edge/long-pass filters, elastically scattered photons are removed. The remaining inelastically scattered photons are directed into the spectrometer, where photons are dispersed on a grating according to their energies prior to their appearance on the CCD. Typically, researchers use near-Infrared (near-IR; 785 and 830 nm) laser sources for RS on biological species (Vallejo-Pérez et al., 2016; Farber et al., 2019b, 2020c; Mandrile et al., 2019; Sanchez et al., 2019c, 2020e). This wavelength choice is based on a phenomenon that is known as “biological window.” A light of a red-near-IR part of the electromagnetic spectrum penetrates deeper into biotical species compared to the blue-green light. Near-IR excitation is also unlikely in the case of photodegradation and thermal degradation of biological specimens. For instance, Kurouski group demonstrated a lack of photodegradation and thermal degradation of a plant leaf upon the use of nearly 0.5 W of 830 nm laser (Sanchez et al., 2019c). It should be noted that the use of green (532 nm) and IR (1,064 nm) excitations in the plant research also has been demonstrated (Yeturu et al., 2016; Altangerel et al., 2017; Egging et al., 2018; Farber and Kurouski, 2018).

## Spectral Treatment and Interpretation of Vibrational Bands

**Figure 3:**
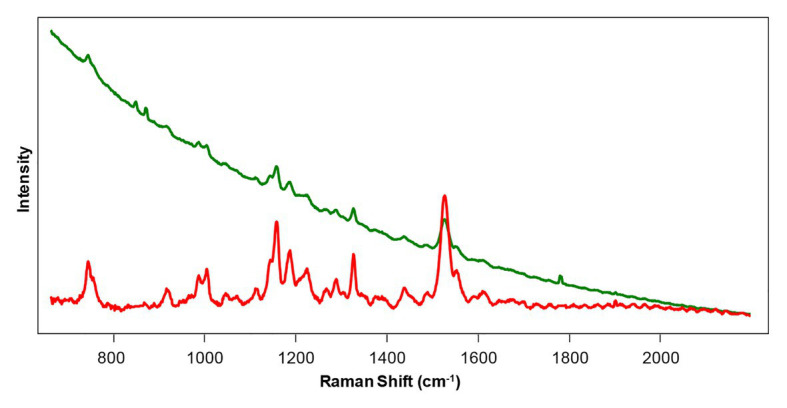
Raw (green) and baseline-corrected (red) Raman spectra collected from a rose leaf with 785 nm excitation.

Raman spectra collected from plant leaves with both 532 nm and 785–830 nm excitations typically have a fluorescence background (Figure 3). Subtraction of such a background is a straightforward process that can be performed either in Matlab (Sikirzhytski et al., 2012) or directly by the spectrometer (Farber and Kurouski, 2018; Farber et al., 2019b).

Direct comparison of Raman spectra can be a challenging task, primarily because the overall spectral intensity can vary with coloration of the analyzed specimen. For instance, Krimmer and co-workers found that dark maize kernels absorbed more and consequently scattered less light relative to the yellow or pale color kernels (Krimmer et al., 2019). Since RS is based on inelastic light scattering, the researchers concluded that dark color maize varieties would produce less intense Raman spectra (under the same experimental conditions) compared to the light color maize varieties. Therefore, observed variations in spectral intensities are likely to originate from different light absorption and scattering properties of such kernels. Kurouski group proposed to solve this problem using normalization. It should be noted that spectra normalization on one particular band that can be assigned to a specific class of molecules, such as carbohydrates, is not appropriate. Such normalization would bias spectral interpretation in regard to the nutrient content of that class of molecules. At the same time, there are several vibrational bands that originate from aliphatic (CH_2_) vibrations, such as 1,440 and 1,458 cm^−1^. Normalization of spectra on these vibrational bands can be used for an unbiased comparison of Raman spectra collected from both leaves and seeds (Farber et al., 2019b, 2020c; Krimmer et al., 2019; Sanchez et al., 2019b,c, 2020b,e). Such normalization allows for avoiding artificial differences in spectra associated with different coloration of analyzed plant material.

Interpretation of vibrational bands in Raman spectra of plant material is a challenging process. In the Raman spectra collected from plant leaves, vibrational bands originating from pectin, cellulose, phenylpropanoids, proteins, and carotenoids can be detected (Table 1).

**Table 1:** Vibrational bands and their assignments for spectra collected from plant leaves and seeds.

Band (cm^−1^)	Vibrational mode	Assignment
480	C–C–O and C–C–C Deformations; Related to glycosidic ring skeletal deformations δ(C–C–C) + τ(C–O) Scissoring of C–C–C and out-of-plane bending of C–O	Carbohydrates (Almeida et al., 2010)
520	ν(C–O–C) Glycosidic	Cellulose (Edwards et al., 1997; Pan et al., 2017)
747	γ(C–O–H) of COOH	Pectin (Synytsya et al., 2003)
849–853	(C_6_–C_5_–O_5_–C_1_–O_1_)	Pectin (Engelsen and Nørgaard, 1996)
917	ν(C–O–C) In plane, symmetric	Cellulose and phenylpropanoids (Edwards et al., 1997)
964–969	δ(CH_2_)	Aliphatics (Yu et al., 2007; Cabrales et al., 2014)
1,000–1,005	In-plane CH_3_ rocking of polyene aromatic ring of phenylalanine	Carotenoids (Schulz et al., 2005) and protein
1,048	ν(C–O) + ν(C–C) + δ(C–O–H)	Cellulose and phenylpropanoids (Edwards et al., 1997)
1,080	ν(C–O) + ν(C–C) + δ(C–O–H)	Carbohydrates (Almeida et al., 2010)
1,115–1,119	Sym ν(C–O–C), C–O–H bending	Cellulose (Edwards et al., 1997)
1,155	C–C Stretching; v(C–O–C), v(C–C) in glycosidic linkages, asymmetric ring breathing	Carotenoids (Schulz et al., 2005) and carbohydrates (Wiercigroch et al., 2017)
1,185	ν(C–O–H) Next to aromatic ring + σ(CH)	Carotenoids (Schulz et al., 2005)
1,218	δ(C–C–H)	Carotenoids (Schulz et al., 2005), xylan (Agarwal, 2014)
1,265	Guaiacyl ring breathing, C–O stretching (aromatic); –C〓C–	Phenylpropanoids (Cao et al., 2006), unsaturated fatty acids (Jamieson et al., 2018)
1,286	δ(C–C–H)	Aliphatics (Yu et al., 2007)
1,301	δ(C–C–H) + δ(O–C–H) + δ(C–O–H)	Carbohydrates (Cael et al., 1975; Almeida et al., 2010)
1,327	δCH_2_ Bending	Aliphatics, cellulose, and phenylpropanoids (Edwards et al., 1997)
1,339	ν(C–O); δ(C–O–H)	Carbohydrates (Almeida et al., 2010)
1,387	δCH_2_ Bending	Aliphatics (Yu et al., 2007)
1,443–1,446	δ(CH_2_) + δ(CH_3_)	Aliphatics (Yu et al., 2007)
1,515–1,535	–C〓C– (in plane)	Carotenoids (Rys et al., 2014; Adar, 2017; Devitt et al., 2018)
1,606–1,632	ν(C–C) Aromatic ring + σ(CH)	Phenylpropanoids (Agarwal, 2006; Kang et al., 2016)
1,654–1,660	–C〓C–, C〓O Stretching, amide I	Unsaturated fatty acids (Jamieson et al., 2018) and proteins (Devitt et al., 2018)
1,682	COOH	Carboxylic acids (Sanchez et al., 2020d)
1,748	C〓O Stretching	Esters, aldehydes, carboxylic acids and ketones (Colthup et al., 1990)

Information provided by Table 1 suggests that RS can be used for the analysis of a large spectrum of compounds in both plant leaves and seeds. It should be noted that an interpretation of spectroscopic changes on the level of molecular species is not always feasible. Nevertheless, RS can be used to probe changes in the most important classes of molecules, such as carotenoids and phenylpropanoids.

## Elucidation of Metabolomic Changes that are Taking Place Upon Bacterial Diseases in Plants

Raman spectroscopy is a non-invasive and non-destructive analytical technique that can be used to reveal the chemical structure and composition of analyzed samples (Kurouski et al., 2015). Unlike IR spectroscopy, RS can be used for the analysis of hydrated biological specimens such as cells and tissues because water provides very low Raman signal (Farber et al., 2019c). Various advantages of RS make this technique the perfect method for the detection of both biotic and abiotic stresses in living organisms, particularly in plant pathology (Egging et al., 2018; Farber and Kurouski, 2018; Farber et al., 2019b, 2020c; Sanchez et al., 2019a,b,c, 2020e). There have been many recent findings on RS breakthroughs in the detection of abiotic and biotic stresses. These include the detection of bacterial infections, secondary diseases, insect infestations, fungal infections, and a variety of other pathogens (Egging et al., 2018; Farber and Kurouski, 2018; Farber et al., 2019b, 2020c; Sanchez et al., 2019a,b,c, 2020e). Although RS can detect pathogens directly (Gan et al., 2017), the below discussed diagnostic of plant biotic and abiotic stresses is achieved by the detection and identification of pathogen-induced changes in the plant biochemistry. Detected changes in the plant metabolism that are taking place on the below discussed diseases are summarized in the Table 2.

**Table 2:** Summary of observed spectroscopic and corresponding biochemical changes in plants that are associated with certain diseases.

Disease	Plant/Organ	Peaks with increase in intensity	Peaks with decrease in intensity	Conclusion
Liberibacter disease in tomatoes (Sanchez et al., 2020b)	Tomato, leaf	-	747 cm^−1^ (pectin); 1,000, 1,115, 1,155, 1,184, 1,218, and 1,525 cm^−1^ (carotenoids)	Liberibacter disease in tomatoes is associated with degradation and fragmentation of host carotenoids and pectin
Huanglongbing (HLB) or citrus greening (Sanchez et al., 2019c)	Orange and grapefruit, leaves	1,601–1,630 cm^−1^ (phenylpropanoids); 1,440–1,455 cm^−1^ (aliphatic)	1,184 and 1,218 cm^−1^ (xylan, carotenoids); 1,525 cm^−1^ (carotenoids), as well as 1,288 cm^−1^ (aliphatic); 1,155 and 1,326 cm^−1^ (cellulose)	HLB is associated with an increase in phenylpropanoids and a decrease in xylan, carotenoids and cellulose
Nutrient deficiency (ND) in citrus trees (Sanchez et al., 2019c)	Orange and grapefruit, leaves	1,247, 1,601–1,630 cm^−1^ (phenylpropanoids); 1,440–1,455 cm^−1^ (aliphatic)	1,184 and 1,218 cm^−1^ (xylan and carotenoids)	ND is associated with an increase in phenylpropanoids
Canker (Sanchez et al., 2019b)	Orange, leaf	-	1,601–1,630 cm^−1^ (phenylpropanoids)	Canker is associated with a decrease in phenylpropanoids content
HLB and blight (Sanchez et al., 2019b)	Orange, leaf	-	-	Diagnostics was achieved *via* the use of partial least square discriminant analysis (PLS-DA)
Ergot (Egging et al., 2018)	Wheat, grain	1,650 and 1,667 cm^−1^ (proteins)	-	Ergot infection may be associated with the expression and deposition of alpha-helical and beta-sheet proteins
Black tip (Egging et al., 2018)	Wheat, grain	1,348 cm^−1^ (monomeric sugars) and 1,600 cm^−1^ (lignin); shift of 862 peak to 856 cm^−1^ (pectin)	862 and 937 cm^−1^ (starch)	Black tip may degrade lignin and ferment starch into monomeric sugars, esterification of pectin
Mold (Egging et al., 2018)	Sorghum, grain	shift of 856 peak to 862 cm^−1^ (pectin); change in ratio between 1,518 and 1,541 cm^−1^ peaks (carotenoids)	1,600 and 1,630 cm^−1^ (phenylpropanoids)	Degradation of phenylpropanoids: a decrease in methylesterfication of pectin caused by the infections suggest a decrease in the length of conjugated double bonds of carotenoids
Ergot (Egging et al., 2018)	Sorghum, grain	1,150, 940, 1,124, and 1,083 cm^−1^ (monomeric sugars); shift of 856 peak to 862 cm^−1^ (pectin); change in ratio between 1,518 and 1,541 cm^−1^ peaks (carotenoids)	1,600 and 1,630 cm^−1^ (phenylpropanoids)	Ergot hydrolyzes starches to produce monomeric sugars: a decrease in methylesterfication of pectin caused by the infections suggest a decrease in the length of conjugated double bonds of carotene
*Fusarium* spp. (Farber and Kurouski, 2018)	Maize, grain	1,658 cm^−1^ (protein); 1,153 cm^−1^ (starch)	1,600 and 1,633 cm^−1^ (phenylpropanoids); 1,547 cm^−1^ (shifted from 1,523 cm^−1^ in healthy) species (carotenoids)	*Fusarium* infection is associated with degradation of phenylpropanoids and deposition of protein in maize kernels; pathogen converts monomeric sugars polymeric carbohydrates
*Aspergillus flavus* (Farber and Kurouski, 2018)	Maize, grain	1,003–1,115 cm^−1^ (monomeric sugars); 1,600–1,633 cm^−1^ (phenylpropanoids)	1,600 and 1,633 cm^−1^ (phenylpropanoids); 1,547 cm^−1^ (shifted from 1,523 cm^−1^ in healthy) species (carotenoids); 1,153 cm^−1^ (starch)	*A. flavus* is associated with a breakdown maize starch into monomeric sugars
*Aspergillus niger* (Farber and Kurouski, 2018)	Maize, grain	1,153 cm^−1^ (starch); 1,600–1,633 cm^−1^(phenylpropanoids)	1,600 and 1,633 cm^−1^ (phenylpropanoids); 1,547 cm^−1^ (shifted from 1,523 cm^−1^ in healthy) species (carotenoids)	*A. niger* converts monomeric sugars polymeric carbohydrates
*Diplodia* spp. (Farber and Kurouski, 2018)	Maize, grain	1,003–1,115 cm^−1^ (monomeric sugars)	1,153 cm^−1^ (starch)	Diplodia is associated with a breakdown maize starch into monomeric sugars
*Abutilon mosaic virus* (Yeturu et al., 2016)	*Abutilon hybridum*, leaf	1,605–1,629 cm^−1^ (phenylpropanoids); 1,440–1,460 cm^−1^ (aliphatic)	-	*Abutilon mosaic virus is associated with an increase in* phenylpropanoids in *A. hybridum*
Tomato yellow leaf curl Sardinia virus (TYCLSV; Mandrile et al., 2019)	Tomatoes, leaf	1,608 cm^−1^ (phenolic); 1,483 cm^−1^ (aliphatic)	1,526 cm^−1^ (carotenoids); 1,420 and 1,483 cm^−1^ (aliphatic), 1,500 and 1,608 cm^−1^ (phenolic); 1,353 cm^−1^ (unidentified);	Small changes in plant biochemistry
Tomato spotted wilt virus (TSWV; Mandrile et al., 2019)	Tomatoes, leaf	1,608 cm^−1^ (phenolic); 1,438 cm^−1^ (aliphatic); 1,353 cm^−1^ (unidentified);	1,483 cm^−1^ (aliphatic)	Small changes in plant biochemistry
Barley yellow dwarf virus (BYDV; Farber et al., 2020a)	Wheat, leaf	1,601–1,630 cm^−1^ (phenylpropanoids)	1,000, 1,115, 1,156, 1,186, 1,218, and 1,525 cm^−1^ (carotenoids)	BYDV is associated with an increase in phenylpropanoids and decrease in carotenoids
Wheat streak mosaic virus (WSMV; Farber et al., 2020a)	Wheat, leaf	1,601–1,630 cm^−1^ (phenylpropanoids)	1,000, 1,115, 1,156, 1,186, and 1,218 cm^−1^ (carotenoids)	WSMV is associated with an increase in phenylpropanoids and decrease in carotenoids

Recently, Kurouski group showed that RS can be used to detect Liberibacter disease in tomatoes (Sanchez et al., 2020c). Liberibacter is a bacterium that infects tomatoes and potatoes worldwide (Glynn et al., 2012; Lin et al., 2012; Nelson et al., 2013; Haapalainen et al., 2018; Swisher Grimm and Garczynski, 2019). While infected plants exhibit observable characteristics, such as chlorosis, stunting, leaf cupping, and scorching, the conventional testing technique of PCR cannot detect the pathogen in the early infection stages at which no symptoms are evident (Liefting et al., 2009; Tamborindeguy et al., 2017). Sanchez and co-workers reported 80% accurate diagnostics of Liberibacter disease on the early infection stage before the development of observable symptoms (Sanchez et al., 2020c). They also found that Raman spectra collected from leaves of Liberibacter-infected tomatoes exhibited lower intensities of carotenoid vibrations compared to healthy tomato plants. This finding suggests that Liberibacter disease in tomatoes is associated with a degradation and fragmentation of host carotenoids. The decrease in the carotenoid content can be also attributed to their conversion to apocarotenoids, signaling molecules that are synthesized by plants upon the development of the stress response. Lee and co-workers found a decrease in the content of pectin in Liberibacter-infected tomatoes. This could be explained by bacteria-driven hydrolysis of pectin, as these molecules are a good source of carbohydrates for this pathogen. Alternatively, changes in pectin content could be due to plant responses to the bacteria-induced stress.

**Figure 4:**
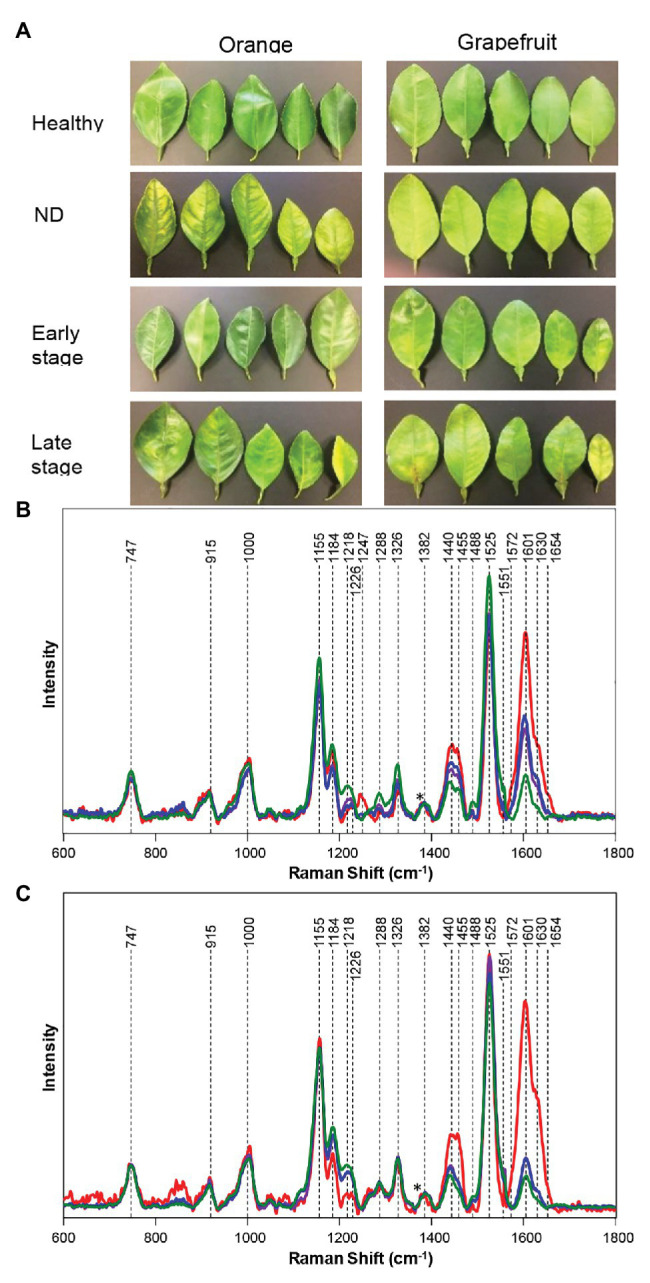
Leaf samples collected from fields for qPCR assay and Raman spectrum **(A)**. Raman spectra generated from leaves of healthy (green), HLB-infected on late (blue) and early (purple) stages, and ND symptoms (red) in grapefruit **(B)**, and orange **(C)** trees. Spectra normalized on cellulose vibrational bands [marked by asterisks (^*^)]. Reproduced with permission from Sanchez et al. (2019c).

Huanglongbing (HLB) or citrus greening is a devastating disease that obliterates citrus trees in Florida and Texas. Kurouski group were able to prove that RS could be used to detect and identify not only HLB but also secondary diseases, such as blight and canker (CA) in HLB-infected orange and grapefruit trees (Sanchez et al., 2019b,c). Sanchez and co-workers also showed that RS could be used to readily diagnoze nutrient deficiencies in these plants (Sanchez et al., 2019c). Sanchez and co-workers collected spectra from four groups of plants: symptomatic qPCR positive plants, and asymptomatic, but qPCR positive plants for HLB, as well as trees that exhibited ND symptoms, which had a similar visual appearance to symptomatic HLB plants, and healthy control plants. In these experiments, leaves were detached from the tree and analyzed immediately using Agilent Resolve spectrometer equipped with 830 nm laser (Figure 1). Although a leaf detachment was not required in this experiment, it was done to minimize exposure to the enormous heat in the area of the spectral analysis (Weslaco, TX). Sanchez and co-workers found that Raman spectra collected from symptomatic and asymptomatic plants exhibited an increase in the intensity of phenylpropanoids (~1,601–1,630 cm^−1^) relative to the intensity of this band in the spectra collected from leaves of healthy trees (negative to HLB by qPCR). It should be mentioned that, in addition to an increase in the intensity of phenylpropanoids, spectra of symptomatic and asymptomatic plants had a decrease in intensities of 1,184 and 1,218 cm^−1^ (xylan), 1,525 cm^−1^ (carotenoids), as well as 1,288 cm^−1^ (aliphatic) and 1,155 and 1,326 cm^−1^ (cellulose) bands (Figure 4).

It has been also found that Raman spectra collected from ND trees had even more intense vibration of phenylpropanoids, together with a band at 1,247 cm^−1^, which was assigned to a phenolic vibration. This evidence allowed for a clear differentiation between HLB, ND, and healthy trees. Sanchez and co-workers also used chemometrics to enable quantitative diagnostics of HLB and ND in citrus trees. In the first set of orthogonal partial least square discriminant analysis (OPLS-DA) models, healthy plants were differentiated from ND and HLB plants. The predicted accuracy was 98% for grapefruit and 87% for orange trees (cross-validation). In the following set of models, chemometrics was used to distinguish symptomatic vs. asymptomatic plants. The accuracy of prediction upon cross-validation appeared to be 100% for grapefruit and 94.4% for orange trees. This work showed that RS can be used for accurate diagnostics of HLB and ND on citrus trees, which helps to enable timely management of that devastating disease in the field. These findings show that non-invasive, non-destructive Raman-based approach allows citrus farmers to properly manage infected trees to increase fruit yield of the rest of their crops.

Microscopic examination of HLB-infected trees confirmed spectroscopic evidence provided by Sanchez and co-workers (Brodersen et al., 2014). It has been found that HLB causes a deformation of cambium cells, has a collapse, callose plug formation, and the thickening of cell walls of parenchyma cells (Brodersen et al., 2014). Cell wall thickening can happen in the attempt to block propagation of bacteria inside the plant. Alternatively, one can imagine that plants secrete low molecular weight phenylpropanoids, aiming to deactivate bacteria. However, these phenylpropanoids later polymerase into high molecular weight phenylpropanoid polymers, also known as lignins. Thus, such phenylpropanoid polymerization can cause histological changes as observed by Brodersen and co-workers.

It should be noted that HLB-infected trees are a subject for secondary infections due to suppressed immune resistance to pathogens. As a result, HLB infected trees become easily susceptible for a blight (BL), one of the most frequently observed secondary diseases on HLB trees, which even further reduces fruit yield and the lifetime of plants. The question to ask was whether RS can be used to differentiate between HLB-infected and HLB + BL plant species. Another question is whether RS can be used to differentiate between HLB and other diseases, such as CA that can appear on citrus trees. Sanchez and co-workers investigated whether RS can be used to differentiate between healthy, HLB, HLB + BL, and orange trees infected by CA (Sanchez et al., 2019b). It has been found that CA and HLB + BL could be detected and identified with 95 and 96% accuracy, respectively. The accuracy of prediction of BL and HLB was 87.7 and 89.4%, respectively. Such a fast and reliable spectroscopic approach is highly important for successful intervention and management of HLB-infected trees.

## Raman-Based Diagnostics of Fungal Diseases in Wheat, Maize, and Sorghum

Kurouski group discovered that RS could also be used to detect fungal infections in wheat and sorghum grain, some of the most economically important food sources grown worldwide (Egging et al., 2018). Pathogens such as ergot, black tip, and mold can cause devastating, up to 50% crop, losses in wheat and sorghum (Egging et al., 2018). Simple diseases, like ergot, are caused by one pathogen. More complex diseases, such as black tip or mold, are caused by several different pathogens co-infecting the plant simultaneously. Egging and co-workers collected Raman spectra from healthy sorghum grain, as well as sorghum grain infected by ergot and mold at different stages of disease proliferation. Spectra were collected form dried grain in the laboratory using Rigaku Progeny spectrometer (Figure 1) equipped with 1,064 nm laser. The researchers also used RS to analyze healthy wheat, wheat black tip, and wheat infected by ergot. It was found that ergot-infected wheat had two distinct peaks at 1,650 and 1,667 cm^−1^ that were not indicated in healthy and black tip-infected wheat. This change in intensity of the amide I region (1,650 and 1,667 cm^−1^) suggests that ergot infection may be associated with the expression and the deposition of alpha-helical and beta-sheet proteins. It was also found that spectra collected from black tip-infected wheat had decreased intensities of bands at 862 and 937 cm^−1^ when compared to healthy wheat spectra. These vibrational bands are associated with C–O–C vibration, which is very typical for starch. In addition, vibrational bands at 1,348 and 1,600 cm^−1^ had increased intensities in black tip-infected wheat when compared to healthy wheat. The 1,348 cm^−1^ band correlates to C–O–H vibration that is common in monomeric sugars. This observation suggests that black tip may ferment starch in wheat into monomeric sugar. The 1,600 cm^−1^ band originates from lignin and suggests that black tip degrades lignin or phenylpropanoid content of the plant. Black tip-infected wheat also has a 856 cm^−1^ peak that is shifted from the regular 862 cm^−1^ peak that healthy and ergot wheat exhibit. The authors proposed that this could be due to methylesterification of pectin caused by the black tip infection. Egging and co-workers used OPLS-DA to enable quantitative prediction of the disease on wheat and sorghum. The researchers found that RS was capable of predicting the diseases on wheat with 100% accuracy (cross-validation; Egging et al., 2018).

Kurouski group also analyzed differences in spectra collected from healthy sorghum, mold sorghum, and ergot sorghum (Egging et al., 2018). It was found that lignin bands at 1,600 and 1,630 cm^−1^ disappeared in mold-infected sorghum, indicating the degradation of lignin associated with mold development. There was also some slight decrease in the intensities of those bands in ergot-infected sorghum when compared to healthy sorghum. Spectra collected from ergot-infected sorghum were also found to have increased intensity at 1,150, 940, 1,124, and 1,083 cm^−1^ bands, indicating that ergot hydrolyzes starches to produce monomeric sugars. Spectra collected from both ergot- and mold-infected sorghum exhibited a shift in their 856 cm^−1^ band to 862 cm^−1^. The authors proposed that this could be due to a decrease in methylesterfication of pectin caused by the infections. Decreases in the methyl-esterified pectin suggests a decreased ability for the grain to resist infection. Finally, changes in ratios between 1,518 and 1,541 cm^−1^ peaks were observed between healthy and infected sorghum. These changes suggest a decrease in the length of conjugated double bonds of carotene. Based on the above-discussed spectroscopic changes, Kurouski group was able to distinguish between mold, ergot, and healthy sorghum using RS with over 96% accuracy.

**Figure 5:**
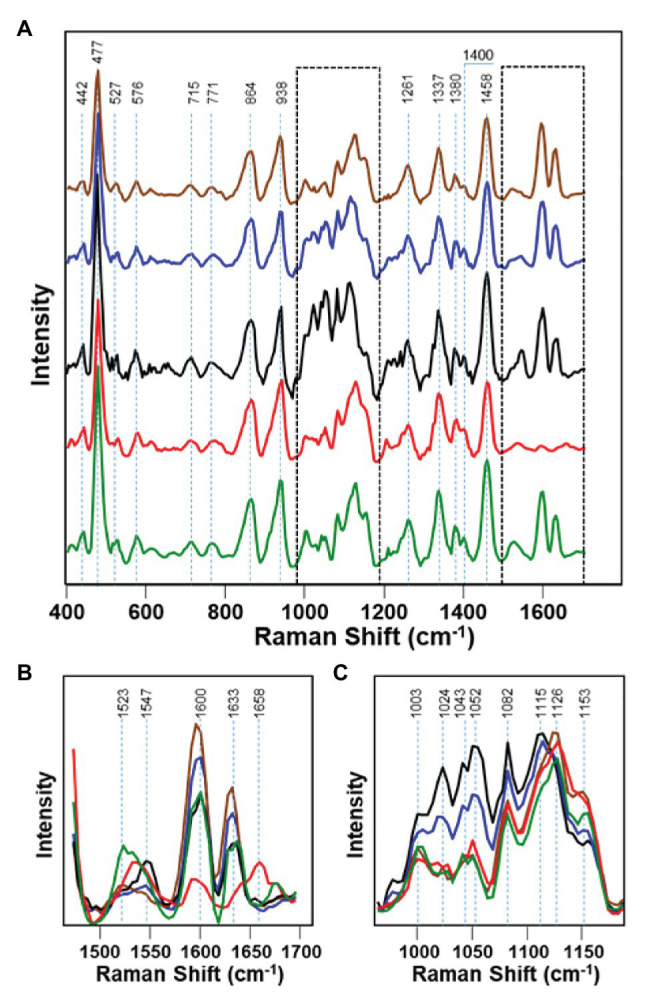
Raman spectra of healthy maize kernels (green) and maize kernels infected by *Aspergillus niger* (brown), *A. flavus* (blue), *Diplodia* spp. (black), and *Fusarium* spp. (red). 1450-1700 cm-1 and 950-1200 spectral regions shown by dashed lines in the panel A are magnified in panels B and C, respectively. Reproduced with permission from Farber and Kurouski, (2018).

Maize, also referred to as corn, is one of the most impactful grains in the world in terms of its uses. With a commercial impact of more than 50 billion in the United States, maize is used as livestock feed, as raw material in the industry, and as a biofuel and serves as a staple for human consumption as food (Farber and Kurouski, 2018). Kurouski group showed that RS could detect fungal pathogens *Aspergillus flavus*, *Aspergillus niger*, *Fusarium* spp., and *Diplodia* spp. in maize with 100% accuracy (Farber and Kurouski, 2018). In this study, Raman spectra were collected from dried grain in the laboratory using Rigaku Progeny spectrometer (Figure 1) equipped with 1,064 nm laser. Healthy maize has vibrational bands attributed to lignin, carbohydrates, proteins, and carotenoids. The 1,600 and 1,633 cm^−1^ bands from lignin completely disappear in *Fusarium*-infected maize, suggesting the significant degradation of lignin (Figure 5). These peaks also had a change in intensity in *A. flavus* and *A. niger*-infected maize, but no noticeable difference in *Diplodia*. Protein exhibits a key vibrational band at around 1,658 cm^−1^ in the *Fusarium*-infected maize, indicating that the growth of this pathogen is strongly associated with the deposition of protein in maize kernels. In healthy maize kernels, carotenoids show an intense peak at 1523 cm^−1^ with another less intense peak at 1547 cm^−1^. *Fusarium*-, *A. flavus*-, and *Diplodia-*infected maize kernels all exhibit a stronger peak at 1547 cm^−1^ rather than 1,523 cm^−1^. This suggests that these pathogens either lead to degradation and fragmentation of carotenoids in maize, produce specific short-chain carotenoids, or convert carotenoids to apocarotenoids. Starch and monomeric sugars are carbohydrates and make up the major components of maize. An increase in C–O–H vibrations were observed in *Diplodia*- and *A. flavus*-infected maize. This suggests that these pathogens breakdown maize starch into monomeric sugars. The authors also observed an increase in the intensity of C–O–C band (1,153 cm^−1^) in the spectra collected from *A. niger*- and *Fusarium*-infected maize, suggesting that these pathogens turn monomeric sugars into polymeric carbohydrates.

## Raman-Based Diagnostics of Viral Diseases

First experimental evidence about a feasibility of Raman-based diagnostics of viruses was provided by Yeturu et al. (Yeturu et al., 2016). The authors demonstrated that the intensity of the collected spectra from *Abutilon hybridum* depends on a degree of the plant infection by *Abutilon mosaic virus*. Expanding upon these findings, Rossi group investigated the accuracy of diagnostics of tomato yellow leaf curl Sardinia virus (TYLCSV) and tomato spotted wilt virus (TSWV) in tomatoes (Mandrile et al., 2019). Using RS and real-time PCR, the researchers monitored inoculated plants over 28 days until the appearance of symptoms. Mandeile and co-workers showed that RS allowed the discrimination of mock inoculated (healthy) from virus-infected specimens with above 70% accuracy after only 14 days after inoculation for TYLCSV and >85% only after 8 days for TSWV. These findings demonstrate a suitability of RS for an early detection of virus infections in tomatoes.

**Figure 6:**
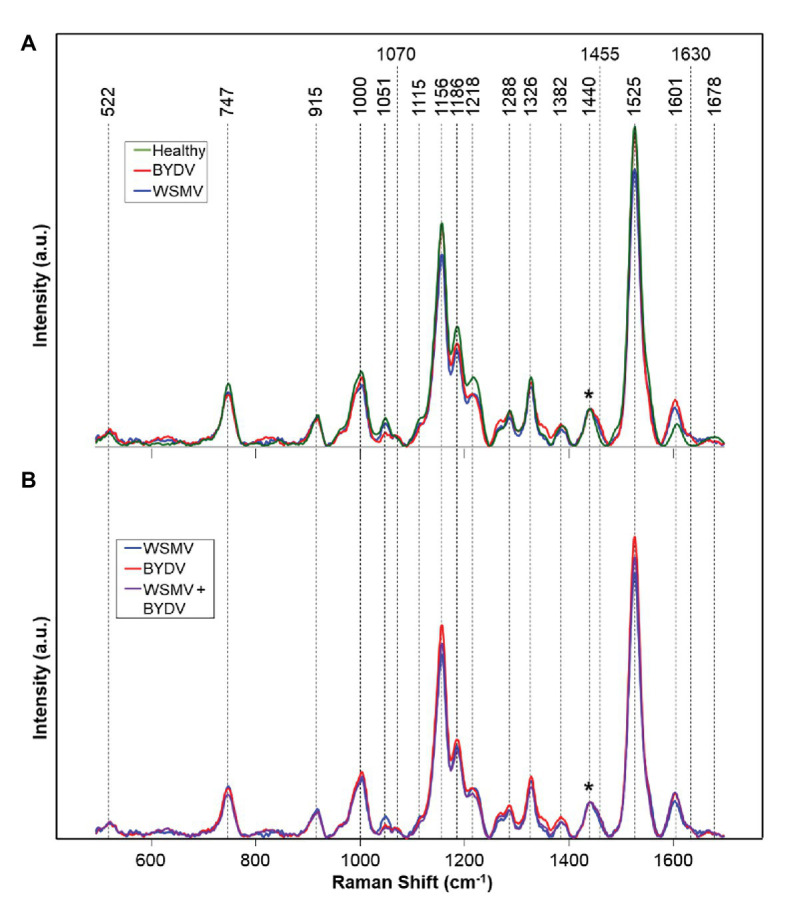
Raman spectra of: **(A)** Healthy and WSMV- or BYDV-infected wheat leaves and **(B)** the combination of these two viruses. Normalization band at 1,440 cm^−1^ is marked by an asterisk (^*^).

Recently, Kurouski and group demonstrated that RS could be used for confirmatory identification of viruses in wheat (Farber et al., 2020a). Farber and co-workers found that RS can be used to differentiate between healthy wheat and wheat infected by wheat streak mosaic virus (WSMV) and barley yellow dwarf virus (BYDV). Lastly, researchers showed that RS could be used to identify whether wheat is infected by these individual viruses or by a combination of WSMV and BYDV, as well as WSMV, BYDV, and Triticum mosaic virus (TriMV; Figure 6).

## Fruit and Seed Quality Control Enabled by Raman Spectroscopy

Tomato is a major fruit, and the need for determining the quality attributes of this fruit in a non-destructive way is in increasing demand. Nikbakht and co-workers proposed to use RS to determine tomato fruit quality (Nikbakht et al., 2011). This study showed that RS could be used to measure important quality parameters of tomatoes such as soluble solid content (SSC), acidity (pH), and color. The work done by Nikbakht and co-workers also showed that RS can be highly effective in quality assessment of both external and internal properties of tomatoes (Nikbakht et al., 2011). Martin and co-workers further expanded the use of RS for the analysis of tomatoes ripening (Martin et al., 2017). The researchers developed a model of tomato ripening based on carotenoid vibrational bands in Raman spectra. Tomato fruits were scanned using bench-top spectrometer equipped with 532 nm laser during their post-harvest time evolution and ripening. It has been found that an increase in carotenoid vibrations were coincident with the onset of the turning stage of the fruit ripening. The model built from the collected data describes the tomato ripening stages and helps to accurately assess postharvest fruit quality control (Martin et al., 2017).

Expanding upon these results, Nekvapil and co-workers investigated the applicability of RS for quality control of fruits (Nekvapil et al., 2018). Nekvapil and co-workers were able to show that RS can be used to scan for fruit freshness, particularly in citrus. By scanning the peels of citrus fruits, such as mandarin oranges, tangerines, and clementines, it was found that the intensity of carotenoids in a fruit can be used to determine the freshness of a fruit. The researchers proposed that this approach can be used to increase consumer trust, safety, and satisfaction when purchasing citrus fruits (Nekvapil et al., 2018). Independently, Feng and co-workers used RS to test eight different citrus varieties (Feng et al., 2013). The researchers were able to build a model to distinguish the citrus varieties. This work demonstrates that RS can be used for accurate, rapid, effective identification of citrus varieties and quality assessment for citrus fruits. Further studies on the ability of RS to be used for the purpose of quality assessment was done by Zhu et al. (2018). Lignification in fruits leads to increased fruit firmness and is important to optimize postharvest fruit handling to minimize quality deterioration. Zhu and co-workers were able to come up with a procedure to use Raman to assess fruit lignification (Zhu et al., 2018). By using Raman spectroscopy, lignification of a fruit can be assessed to determine ripeness.

**Figure 7:**
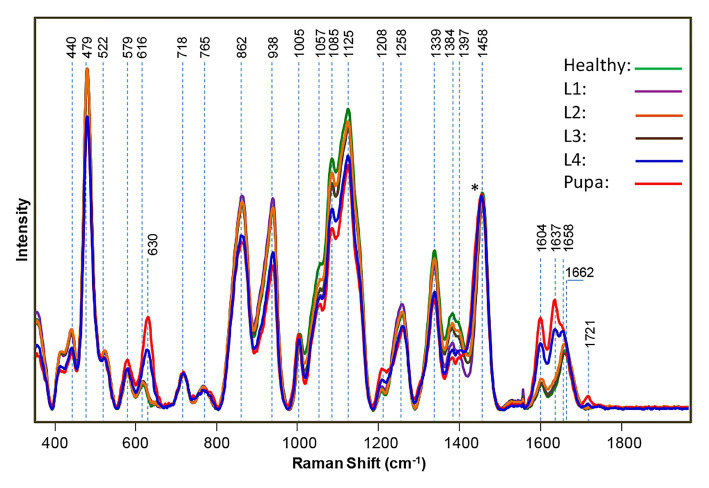
Raman spectra of healthy, uninfested cowpea seeds and seeds infested by bruchids at larval and pupal stages, normalized to the 1,458 cm^−1^ peak [indicated by an asterisk (^*^)]. Reproduced with permission from Sanchez et al. (2019a).

The cowpea bruchid is an insect that damages legumes, such as beans and peas by feeding on them. The bruchid lays its eggs on the seeds, making the detection of infestation a difficult problem. If left unchecked, two bruchids could destroy up to 50% of a ton of harvest cowpea. Sanchez and co-workers discovered that RS could be used for the detection of bruchid larvae as well as their excrements inside intact seeds (Sanchez et al., 2019a). Specifically, Sanchez and co-workers collected spectra from cowpea seeds infested with bruchids. They took the spectra of bruchids at different developmental stages, including the first, second, third, and fourth larvae (L1–L4) or pupa. The respective spectra were then averaged and compared to healthy cowpea seeds. The spectra were normalized on the 1,458 cm^−1^ band. They found in L1–L3 infected seeds that gradual decreases in intensity occurred in (C–O–H) vibrational bands (440, 479, 522, 862, 938, 1,057, 1,085, 1,125, 1,258, 1,339, 1,384, and 1,397 cm^−1^) and observed drastic changes in these bands in L4 and pupa (Figure 7). The differences between healthy, early stage (L1–L3), and late stage (L4 and pupa) infections were statistically significant. The Kurouski group also observed additional changes in the intensity of bands in L4 and pupa spectra. To determine if the spectral changes were from insects feeding on the seeds or from the actual bruchid larvae, Raman spectra were taken from L4 seeds where the larvae were removed and called the L4’ spectrum. They found that the observed spectral changes in the 1,600–1725 cm^−1^ region were due to the larvae.

Using this information, RS can also be used to monitor the growth of insect larvae. The vibrational band at ~630 cm^−1^ was found to be assigned to uric acid and was a major component of the bruchid excrement. A decrease in intensity in all vibrational bands was also observed and associated with carbohydrates in the L4’ spectrum. Using partial least square discriminant analysis (PLS-DA) and cross-validation, Kurouski group was able to determine the early stage infection with 93.7% accuracy, the late stage infection with 100% accuracy, and the healthy stage with 85% accuracy. The results of Lee and co-workers demonstrate that RS can detect insects within plant hosts, such as cowpeas.

Piot and co-workers showed that RS can be used to probe wheat grain to follow the evolution of protein content and structure during grain development (Piot et al., 2002). The work done by Piot and co-workers shows Raman spectroscopy’s ability to not only detect molecular species at the micro scale but also give information on the structure and their binding with neighboring molecules. For example, Piot and co-workers discovered that an increase in *α*-helical protein content occurs when the kernel hardens during grain ripening.

Virgin olive oil is different from other vegetable oils because it is edible from the moment of production. However, olive oil comes in different grades, and if its quality is not high enough, it cannot be considered virgin olive oil and must undergo further refinement prior to consumption. Muik and co-workers were able to use RS to differentiate between olives of different qualities (Muik et al., 2004). Sound olives, olives with frostbite, olives collected from the ground, fermented olives, and olives with diseases were analyzed using RS. Principal component analysis (PCA), hierarchical cluster analysis (HCA), and soft independent modeling of class analogy (SIMCA) were used to analyze differences in vibrational bands. Based on the acquired spectra and the above-discussed statistical approaches, Muik and co-workers were able to identify the type with 95% accuracy for sound olives, 93% accuracy for frostbite, 96% accuracy for ground, and 92% accuracy for fermented olives (cross-validation; Muik et al., 2004). In addition, none of the damaged olive samples were wrongly predicted to the class for sound olives.

## Spectroscopic Identification of Plant Species and their Varieties

**Figure 8:**
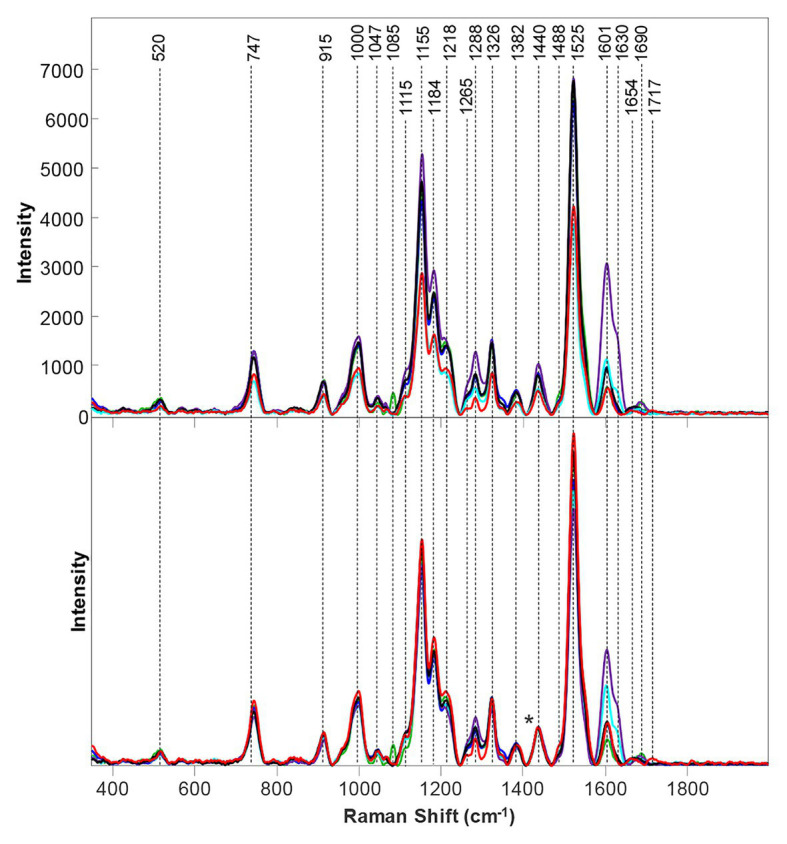
Baseline-corrected (top) and area normalized (bottom) Raman spectra collected from poison ivy (red), palmer amaranth (green), water oak (blue), white crownbeard (marine), buckbrush (purple) and saw greenbrier (black).

Urushiol oils, a mixture of pentadecylcatechols, are responsible for the allergic reactions caused by the notorious poison ivy (Hodgson, 2012). Server rashes, skin inflammation, uncolored bumps, and blistering are some of the common symptoms exhibited by those who were unfortunate enough to come across poison ivy (Yang et al., 2000; Gober et al., 2008; Joly et al., 2019). Because these reactions take hours or days to occur, those covered in urishiol unknowingly spread the substance once they have come in contact with poison ivy. While extensive washing with soap and water may stop the spread of urishiol oils, there is no way to avoid these symptoms other than to avoid contact with poison ivy (Joly et al., 2019). Unfortunately, it is difficult for those without botanical experience to differentiate poison ivy from other plants. To help overcome this problem, Kurouski group developed RS for non-invasive, non-destructive, confirmatory, and label-free identification of poison ivy (Farber et al., 2020b). The exhibited vibrational bands in poison ivy could be assigned to a few key groups: cellulose, pectin, carotenoids, phenylpropanoids, xylan, protein, aliphatic, and carbonyl/ester groups (Figure 8). While some of these bands appear in other similar plants, poison ivy has a unique band at 1,717 cm^−1^, which is not evident in other plants. This unique band, along with other key spectroscopic features in poison ivy’s Raman fingerprint (such as its high carotenoid intensity), can be used for the identification of poison ivy with 100% accuracy when compared to similar looking plants, such as palmer amaranth, water oak, white crown beard, and saw greenbrier (98.2% accuracy when compared to buckbrush).

Potatoes are a staple food for people all over the world because of high starch content, simple cultivation, and high production. Potatoes are made up of about 83% water, 12% carbohydrates, and the remaining 4% includes proteins, vitamins, and other trace elements (Morey et al., 2020). The proportions vary based on the potato type and where it was cultivated. While there are some chemical methods to investigate starch content, these approaches are indirect, destructive, labor consuming, and time consuming. Kurouski group was able to use RS to asses nutrient content of potato tubers (Morey et al., 2020). In addition, RS can be used to identify nine different potato varieties as well as to determine the origin of cultivation. Using spatially offset Raman spectroscopy (SORS), Kurouski group found that a peak intensity varied by potato variety at 479 and 1,125 cm^−1^ for starch, 1,600 and 1,630 cm^−1^ for phenylpropanoid, 1,527 cm^−1^ for carotenoid content, and 1,660 cm^−1^ for protein content. Using these differences in relative intensities and PLS-DA with cross-validation, Kurouski group was able to identify a potato variety, as well as to determine the location of potato cultivation with accuracy ranging from 81 to 100%. In addition, Kurouski group was able to demonstrate that the intensity of the 479 cm^−1^ band (which correlates to starch) linearly increases with an increase in the starch content of the sample (Morey et al., 2020). These results demonstrate that RS can be used for highly accurate determination of the starch content in intact potatoes.

Currently, the identification of specific genotypes can be only accomplished *via* visual recognition of distinct phenotypical appearances (if applicable) or by genotype sequencing. Both have many downfalls. Identifying genotypes by visual recognition is often difficult and requires substantial taxonomic expertise. The results are often subjective and often can be incorrect. Genotype sequencing is destructive, time- and labor-consuming. The answer to these genotype identification problems can be solved by the use of RS (Farber et al., 2020c). Farber and co-workers show that chemometric analysis of peanut leaflet spectra provides an accurate identification of different varieties, genotype, and can be used for the prediction of nematode resistance and oleic-linoleic oil (O/L) ratio (Farber et al., 2020c). Raman-based analysis of seeds provides accurate genotype identification and also can identify carbohydrates, proteins, fiber, and other nutrients obtained from the readings of peanut seeds. Ten different genotypes of peanuts were grown and their leaflets were scanned. They all exhibited similar profiles with vibrational bands being mainly due to carbohydrates, cellulose, pectin, carotenoids, phenylpropanoids, proteins, and carboxylic acid. A PLS-DA model was built, and it was found that Raman could identify peanut variety with 80% accuracy just from scanning leaflets (cross-validation was used). Root-knot nematodes feed on peanut plants and peanut plant resistance is important to peanut cultivators. Kurouski group found that peanut plant resistance was related to changes in bands associated with carotenoid and phenylpropanoid. In addition, peanut cultivators prefer peanuts with high oleic ratios as they have a longer shelf life which leads to reduced rancidity. Also, it has been found that peanuts with high oleic ratios reduce serum cholesterol levels and reduce chances of cardiovascular disease. RS revealed that plants with high oleic ratios have lower phenylpropanoid content whereas all other peaks remained nearly identical. Farber and co-workers found Raman to be 82% accurate in the identification of peanuts with high oleic ratios against those with normal ratios. Raman scanning of seeds was done to see if it was more accurate than scanning leaves of peanut plants. The results show that Raman is 95% accurate in the identification of peanut seeds when compared to the 82% of leaves.

**Figure 9:**
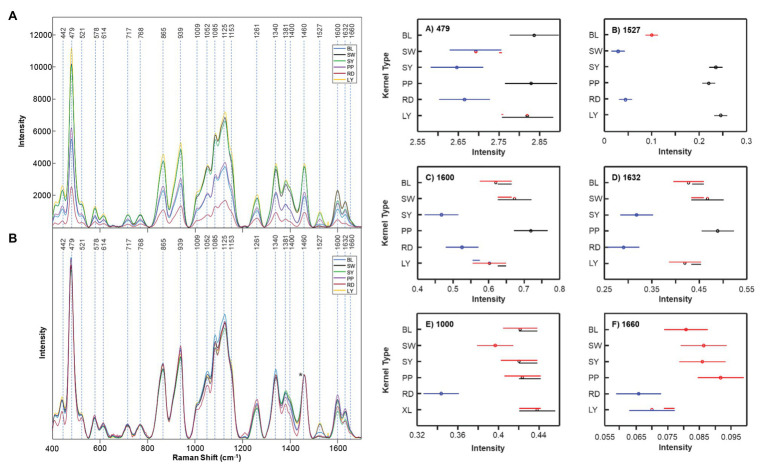
Right: Raw **(A)** and normalized **(B)** Raman spectra of BL, SW, SY, PP, RD, and LY maize kernels. The 1,458 cm^−1^ peak, which was used for spectral normalization, is indicated by an asterisk (^*^). Left: Means (circles) and confidence intervals for the intensities of the maize kernel spectra, normalized to 1,458 cm^−1^, at the indicated wavenumbers. Colors indicate significantly different groups. Multiple colors indicate a member of a group that has an overlap between two separate groups. Reproduced with permission from Krimmer et al. (2019).

Because of the popularity of maize as a food source, further research on the possibilities of Raman and maize was performed by Krimmer and co-workers. The researchers found that RS can be used to access the nutrient content of maize. Specifically, it can predict the content of carbohydrates, fibers, carotenoids, and proteins in six different varieties of maize (Krimmer et al., 2019). To achieve this, Krimmer and co-workers collected more than 600 spectra form six different varieties of maize. All six varieties had similar spectral profiles except the darker kernels scanned had lower intensities. This is because of different light absorption and scattering properties of these maize kernels affect the scan. This problem can be solved by normalization, particularly at the 1,458 cm^−1^ peak which all spectra display. The authors analyzed the intensities of bands at 479 cm^−1^ (starch), 1,530 cm^−1^ (carotenoids), 1,600/1,632 cm^−1^ (both fiber), and 1,640–1,670 cm^−1^ (protein region) to quantify the content of carbohydrates, carotenoids, fibers, and proteins in maize (Figure 9). In addition, Krimmer and co-workers showed that RS in combination with chemometric methods can be used for highly accurate typing of maize varieties.

## Non-Invasive Assessment of Cannabinoid Content in Plants

**Figure 10:**
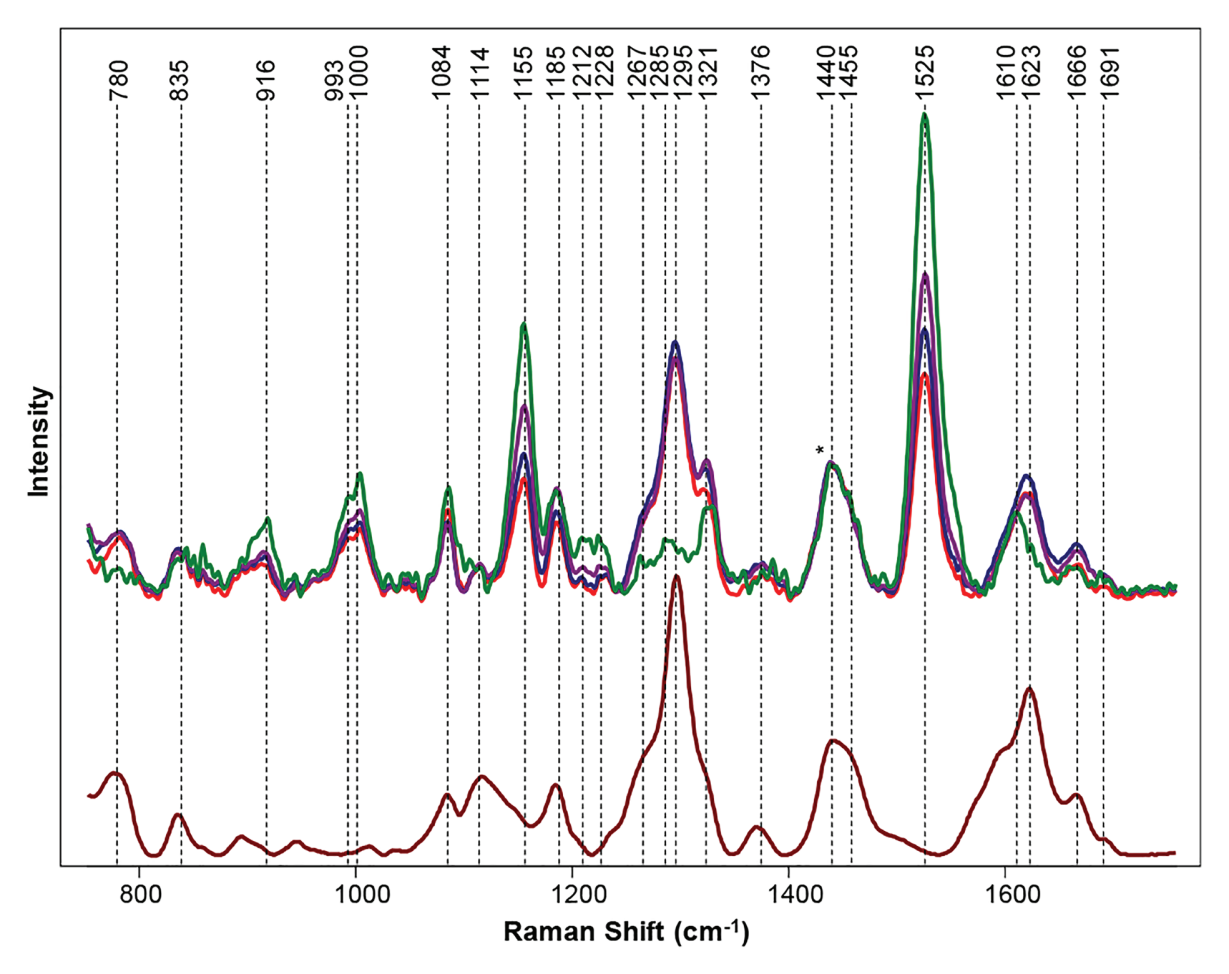
Top: Raman spectra collected from hemp (green), GC (purple), TCC (blue), and TS (red). Bottom: Raman spectrum of THCA extract (maroon). Spectra normalized on CH_2_ vibrations (1,440 and 1,455 cm^−1^) are present in nearly all classes in biological molecules [marked by asterisks (^*^)]. Reproduced with permission from Sanchez et al. (2020d).

Hemp has been used to treat pain since 2,900 B.C. and has pharmacological effects from a variety of cannabinols (Hartsel et al., 2016). While there are over 100 different cannabinoids that can be isolated from cannabis plants, clear physiological effects have only been determined for a few such as delta-9 tetrahydrocannabinol (THC), cannabidiol (CBD), and cannabigerol (CBG; Appendino et al., 2008; Borrelli et al., 2013). THC is illegal, but CBD and CBG are legal and have been shown to reduce chronic pain, inflammation, anxiety, and depression. Cannabis is consumed by 147 million people, which is about 2.5% of the world population (Sanchez et al., 2020d). Cannabis is a hemp plant that contains tetrahydrocannabinolic acid (THCA) in amounts higher than industrial hemp. This THCA is the source of the psychoactive THC that forms from its oxidation. As the most widely cultivated and trafficked illicit drug in the world, it requires substantial effort from border control and law enforcement to control illegal trafficking. Cultivation of cannabis plants with large amounts of CBD and CBG, simultaneously exhibiting little to no THC, would be ideal for growers. Currently, high performance liquid chromatography (HPLC) can be used to determine the amount of cannabinoids in plant material, but the process is non-portable, destructive, and time/labor consuming (Patel et al., 2017; Zivovinovic et al., 2018; Burnier et al., 2019; Nie et al., 2019). Sanchez and co-workers showed that RS can be used to differentiate between hemp, cannabis, and CBD-rich hemp with 100% accuracy using OPLS-DA with cross-validation (Sanchez et al., 2020d). Using a handheld Raman spectrometer, the spectrum of hemp were found to have vibrational bands from cellulose, carotenoids, and lignin. Multiple varieties of cannabis were scanned, and all clearly demonstrated a presence of THCA with key peaks at 780, 1,295, 1,623, and 1,666 cm^−1^ (Figure 10). It was also found that vibrational bands assigned to carotenoids had higher intensity in hemp scans relative to scans of cannabis. This result indicates that hemps have higher carotenoid content compared to cannabis. Similarly, higher intensity in cellulose peaks in hemp indicate a higher amount of cellulose in hemp when compared to cannabis. Using the information gathered by Sanchez and co-workers, a model was set up to determine if Raman spectrometry could be used to differentiate between hemp and cannabis. The result was 100% accuracy in classification between hemp and cannabis. Sanchez and co-workers were also able to detect THCA in intact growing plants because of the vibrational band at 1,691 cm^−1^ that correlates to the carboxyl group of THCA. Therefore, Sanchez and co-workers showed that RS can predict the amount of THC in a sample without necessary oxidation of THCA to THC (Sanchez et al., 2020d). In another study, Sanchez and co-workers took this idea further and were able to detect other cannabinoids, such as CBD, CBG, CBGA, and CBDA on top of THCA and THC (Sanchez et al., 2020a). These discoveries allowed the Kurouski lab to not only differentiate hemp vs. cannabis but also detect CBD-rich hemp with 100% accuracy [63]. Their extensive analysis of Raman spectra on the six major cannabinoids (THC, THCA, CBD, CBDA, CBG, and CBGA) allows for the differentiation between THC/THCA vs. CBD/CBDA vs. CBG/CBGA and can be used to identify cannabis variety with 97% accuracy (Sanchez et al., 2020a).

## Future Perspectives

Research articles published over the last decade provided strong experimental evidence of high sensitivity of RS in determination of changes in plant biochemistry that are associated with biotic and abiotic stresses (Yeturu et al., 2016; Egging et al., 2018; Farber and Kurouski, 2018; Farber et al., 2019b; Mandrile et al., 2019; Sanchez et al., 2019a,b,c). These studies also showed that plant biochemistry uniquely changes as a result of such stresses (Egging et al., 2018; Farber and Kurouski, 2018; Mandrile et al., 2019; Sanchez et al., 2019c). This allows for the use of RS in diagnostics of biotic and abiotic stresses in plants. This high sensitivity to small changes in plant biochemistry also enables Raman-based identification of plant species and their varieties, as well as allows for Raman-based selection and breeding of plants (Krimmer et al., 2019; Farber et al., 2020b,c; Sanchez et al., 2020a,d). Although to date, most of the reported experiments were made in greenhouses (Altangerel et al., 2017; Mandrile et al., 2019; Sanchez et al., 2020b,c), there is a growing body of evidence about successful use of RS in the field (Sanchez et al., 2019b,c, 2020e; Farber et al., 2020a). Once this practice will become the routine of research studies – recognition of RS as a reliable agricultural method will tremendously increase.

The use of RS in agriculture can be further empowered by direct elucidation of biochemical changes that are taking place upon the above-discussed biotic and abiotic stresses. Mass spectroscopy (MS) and HPLC coupled to MS are excellent analytical techniques for analytical characterization of changes in plant biochemistry (Hijaz et al., 2013; Killiny and Nehela, 2017; de Moraes Pontes et al., 2020). Their use will allow for the determination of biochemical origin of the observed spectroscopic changes on the level of molecular analytes. The potential of RS can be further enchanted by its coupling to already established imaging (Mahlein et al., 2012; Mutka and Bart, 2015) and molecular techniques (Schaad and Frederick, 2002; Liu et al., 2015; Zhang et al., 2017). For instance, quick surveillance of large field territories by RGB, thermography or hyperspectral imaging can be used to navigate RS to the “danger” or “problematic” areas (Gowen et al., 2007; Mahlein et al., 2012; Raza et al., 2015). Such UAV-guided RS-based approaches can save enormous resources in diagnostics of biotic and abiotic stresses. This approach can be used to overcome the existing low-throughput of RS. Specifically, the use of hand-held spectrometers requires a direct contact with the analyzed plant that substantially reduces the analysis of large agricultural territories even with 1 s acquisition time that is currently required for such diagnostics. Also, in the light of numerous diseases simultaneously present on a plant, RS can be considered as a “fast screening” approach that may be used for rapid screening of plants. If more sophisticated or accurate analysis is needed, molecular methods of analysis, such as PCR, qPCR, or ELISA, can be used (Clark and Adams, 1977; Schaad and Frederick, 2002; Liu et al., 2015; Zhang et al., 2017).

Substantial limitation of broad utilization of commercially available hand-held spectrometers is their relatively high cost (~$30,000–$60,000). This will likely limit the possession of instruments by individual farmers. One can expect that continuous technological development of spectrometers that enabled their militarization will also reduce the cost of these devices in the nearest future. Nevertheless, the use of RS in agriculture is likely to be implemented as a service in which a farmer can order a non-invasive and reagent-free scan of the field to detect the presence of biotic and abiotic stresses. Collected spectra can be transferred to a server for the analysis using Bluetooth or WiFi or analyzed directly in the field by the hand-help instrument. Next, the farmer will receive information about the status of the field together with GPS coordinates of the analyzed locations.

## Conclusion

This review shows the potential for RS in the future of digital farming. Raman spectrometry’s portable and quick analysis allows for timely detection of biotic and abiotic stresses in plants. In addition, Raman can be used as an advanced method in plant breeding and selection thanks to being both non-invasive and non-destructive. Furthermore, RS can be used for plant phenotyping and nutrient analysis. The benefits of RS will surely become more clear to others and the adoption of Raman spectrometry in digital farming will become more common.

## Author Contributions

WP review of previously reported results and systematization of literature reports. DK methodology, funding acquisition, and supervision. Both the authors contributed to the article and approved the submitted version.

### Conflict of Interest

The authors declare that the research was conducted in the absence of any commercial or financial relationships that could be construed as a potential conflict of interest.
